# Contactin-1 Is Reduced in Cerebrospinal Fluid of Parkinson’s Disease Patients and Is Present within Lewy Bodies

**DOI:** 10.3390/biom10081177

**Published:** 2020-08-12

**Authors:** Madhurima Chatterjee, Inger van Steenoven, Evelien Huisman, Linda Oosterveld, Henk Berendse, Wiesje M. van der Flier, Marta Del Campo, Afina W. Lemstra, Wilma D. J. van de Berg, Charlotte E. Teunissen

**Affiliations:** 1 Neurochemistry Laboratory, Clinical Chemistry Department, Amsterdam Neuroscience, Amsterdam University Medical Centers, Vrije Universiteit, 1105 AZ Amsterdam, The Netherlands; madhurima.cob@gmail.com (M.C.); m.delcampomilan@amsterdamumc.nl (M.D.C.); 2 Alzheimer Center Amsterdam, Department of Neurology, Amsterdam Neuroscience, Amsterdam University Medical Centers, Vrije Universiteit, 1105 AZ Amsterdam, The Netherlands; i.vansteenoven@amsterdamumc.nl (I.v.S.); wm.vdflier@amsterdamumc.nl (W.M.v.d.F.); a.lemstra@amsterdamumc.nl (A.W.L.); 3 Department of Anatomy and Neurosciences, Section Clinical Neuroanatomy, Amsterdam Neuroscience, Amsterdam University Medical Centers, Vrije Universiteit, 1105 AZ Amsterdam, The Netherlands; e.timmermans@amsterdamumc.nl (E.H.); l.oosterveld@amsterdamumc.nl (L.O.); wdj.vandeberg@amsterdamumc.nl (W.D.J.v.d.B.); 4 Department of Neurology, Amsterdam Neuroscience, Amsterdam University Medical Centers, Vrije Universiteit, 1105 AZ Amsterdam, The Netherlands; h.berendse@amsterdamumc.nl; 5 Department of Epidemiology & Biostatistics, Amsterdam Neuroscience, Amsterdam University Medical Centers, Vrije Universiteit, 1105 AZ Amsterdam, The Netherlands

**Keywords:** contactin, Lewy bodies, cerebrospinal fluid (CSF), biomarker, synaptic degeneration, Parkinson’s disease (PD), dementia with Lewy bodies (DLB)

## Abstract

Synaptic degeneration is an early phenomenon in Parkinson’s disease (PD) pathogenesis. We aimed to investigate whether levels of synaptic proteins contactin-1 and contactin-2 in cerebrospinal fluid (CSF) of PD patients are reduced compared to dementia with Lewy bodies (DLB) patients and controls and to evaluate their relationship with α-synuclein aggregation. Contactin-1 and -2 were measured in CSF from PD patients (*n =* 58), DLB patients (*n =* 72) and age-matched controls (*n =* 90). Contactin concentration differences between diagnostic groups were assessed by general linear models adjusted for age and sex. Contactin immunoreactivity was characterized in postmortem substantia nigra, hippocampus and entorhinal cortex tissue of PD patients (*n =* 4) and controls (*n =* 4), and its relation to α-syn aggregation was evaluated using confocal laser scanning microscopy. Contactin-1 levels were lower in PD patients (42 (36–49) pg/mL) compared to controls (52 (44–58) pg/mL, *p =* 0.003) and DLB patients (56 (46–67) pg/mL, *p =* 0.001). Contactin-2 levels were similar across all diagnostic groups. Within the PD patient group, contactin-1 correlated with t-α-syn, tTau and pTau (*r =* 0.30–0.50, *p <* 0.05), whereas contactin-2 only correlated with t-α-syn (*r =* 0.34, *p =* 0.03). Contactin-1 and -2 were observed within nigral and cortical Lewy bodies and clustered within bulgy Lewy neurites in PD brains. A decrease in CSF contactin-1 may reflect synaptic degeneration underlying Lewy body pathology in PD.

## 1. Introduction

Parkinson’s disease (PD) is the most common neurodegenerative disorder after Alzheimer’s disease and its prevalence increases with age [1]. The pathological hallmarks of PD consist of loss of nigro-striatal dopaminergic neurons and intraneuronal Lewy bodies (LBs) and intraneuritic Lewy neurites (LNs), fibrillary aggregates in predilected brain regions [2]. The presynaptic protein alpha-synuclein (α-syn) is the main component of LBs and LNs and is implicated in the pathogenesis of PD [3]. Synaptic protein alteration may result from synaptic α-syn accumulation, leading to synaptic and subsequent axonal damage [4]. Synapse loss [5] and axonal damage [6] are prominent early pathological changes that could contribute to the various motor and non-motor symptoms [7,8] in early-stage PD.

The clinical diagnosis of PD is primarily based on the presence of Parkinsonism. In addition, PD patients experience a wide range of non-motor symptoms, including sleep, as well as autonomic, psychiatric and cognitive dysfunction. There is a large variability in disease-onset and progression among PD patients [8]. Early diagnosis of PD is needed to provide an early window for therapeutic intervention aiming at slowing down or halting disease development. Therefore, there is a strong need for cerebrospinal fluid (CSF) biomarkers to support an earlier diagnosis and detect underlying pathogenic mechanisms in PD. Promising biomarker candidates for early diagnosis of PD that have emerged are: alpha-synuclein species (total α-syn, oligomeric α-syn and phosphorylated α-syn), β-amyloid 1–42, axonal damage markers (tau, neurofilament light (NfL)) and lysosomal enzymes [9,10,11]. However, the overlap in CSF levels of these biomarkers between PD and controls indicates that additional biomarkers are needed, which should be combined to fully capture the different underlying pathologies of PD [11,12].

Since synaptopathy and axonal degeneration are early events in PD [6,13,14,15,16,17], biofluid biomarkers that mirror synaptic and axonal degeneration may hold potential as early diagnostic markers for PD. Contactin-1 and contactin-2 are cell adhesion molecules involved in synaptic plasticity and axonal organization [18]. They are expressed on synaptic membranes and (juxta)paranodal regions of axons [18]. Previous CSF proteomics-based studies showed lower levels of contactin-1 in PD compared to controls and dementia with Lewy bodies (DLB) [19]. To our knowledge, CSF contactin-2 levels have not been investigated in PD patients before, although a proteomics-based study found a tendency towards lower levels of contactin-2 in the post-mortem prefrontal cortex of PD patients, compared to controls [20]. Based on these previous findings, we hypothesized that contactin-1 and -2 might be reduced in CSF of PD patients compared to controls, reflecting synaptic and axonal loss. Since DLB is a synucleinopathy, closely related to PD [21], we additionally investigated contactin levels in CSF of DLB patients. The aims of this study were to investigate (i) whether CSF levels of contactin-1 and contactin-2 are reduced in CSF of PD patients, (ii) whether contactins can discriminate PD patients from controls and DLB patients, and (iii) their relationship with clinical outcome measures of disease severity, and with CSF markers of neurodegeneration α-syn, tTau and pTau, which are also associated with synucleinopathies [22,23]. In addition, we explored whether contactin-1 and -2 are associated with Lewy pathology in post-mortem brain tissue of patients diagnosed with PD.

## 2. Materials and Methods

### 2.1. Subjects

We included PD patients (*n* = 58) based on clinical diagnosis, and age-matched volunteers without neurological symptoms (*n* = 50) from the outpatient clinic for movement disorders of the Amsterdam UMC, location VUmc [24]. Patients with DLB (*n* = 72) and non-demented controls with subjective cognitive decline (SCD) (*n* = 40) were selected from the Amsterdam Dementia Cohort [25]. In total, the control group consisted of 90 subjects. Controls with SCD and healthy volunteers were analyzed as one control group as CSF levels of core Alzheimer’s disease (AD) biomarkers (Aβ42, tTau, pTau), Mini Mental State Examination (MMSE) scores, contactin-1 and contactin-2 levels did not differ (Figure S7).

The demographic details of all the subjects are outlined in Table 1. All subjects provided written informed consent for use of biomaterial and clinical data for research and the study was approved by the local medical ethical review board. The study was conducted according to the revised Declaration of Helsinki and Good Clinical Practice guidelines. The details of clinical diagnosis are provided in Appendix A.

### 2.2. CSF Collection and Assays

CSF was collected by lumbar puncture and stored in polypropylene tubes according to previous published JPND-BIOMARKAPD guidelines until analysis [26]. Commercially available analytically validated ELISAs [27] were used for measuring contactin-1 (ELH-CNTN1-1, RayBiotech, Norcross, GA, USA) and contactin-2 (Cat Nos. DY1714-05, DY008, R&D Systems, Inc., Minneapolis, MN, USA). CSF samples were diluted 1:20 in reagent diluent provided in the kit and the assay was performed according to the manufacturer’s protocols. CSF amyloid beta-42 (Aβ42), total tau (tTau) and phosphorylated tau (pTau) were measured as part of routine diagnosis at the neurochemistry laboratory at Amsterdam University Medical Center, The Netherlands, using commercially available enzyme-linked immunosorbent assay (ELISA; Innotest, Fujirebio, Ghent, Belgium) [28]. Total alpha-synuclein (t-α-syn) was measured in triplicate by time-resolved fluorescence energy transfer (TR-FRET) immunoassay and the mean of the triplicate was used, as previously published [24].

### 2.3. Immunohistochemistry (IHC), Immunofluorescence (IF) and Microscopy

The details of post-mortem brain tissue, IHC protocol and microscopy are provided in Appendix A and Appendix B. Anti-contactin-1 (1:100, cat no. ab66265, Abcam, Cambridge, UK) and anti-Contactin-2 (1:100, HPA001397, Atlas Antibodies, Stockholm, Sweden) were used as primary antibodies for IHC. Labeling of primary antibody was detected using DAKO anti-rabbit/mouse EnVision+ System-HRP (DAKO, 45007, Glostrup, Denmark). Nuclei were visualized by Mayer’s hematoxylin counterstain (Merck, MHS1, Zwijndrecht, The Netherlands). Finally, the slides were mounted with coverslips (Thermo Fisher Scientific Gerhard Menzel B.V. & Co. KG, Braunschweig, Germany) using Entellan mounting medium (cat no. 107960, EMD Millipore, Burlington, MA, USA).

The following primary antibodies were used for evaluation of colocalization using IF between contactin-1/-2 and pathological α-synuclein: rabbit polyclonal anti-contactin-1 (1:40; cat no. ab66265, Abcam, Cambridge, UK), rabbit polyclonal anti-contactin-2 (1:40; HPA001397, Atlas Antibodies, Stockholm, Sweden) in combination with mouse monoclonal anti-p-Ser129-aSyn (11A5 1:30,000, courtesy of Prothena Biosciences [29]). Donkey anti-rabbit IgG Alexa-594 (1:200, cat no. A-21207, Thermo Fisher Scientific, Landsmeer, The Netherlands) and donkey anti-mouse IgG Alexa-488 (1:200; A21202, Mol. Probes, Thermo Fisher Scientific) were used as the secondary antibody. Slides were mounted with coverslips using Mowiol as a mounting medium.

### 2.4. Statistics

The Kolmogorov–Smirnov test was used to check normal distribution of the data. Contactin-1 and contactin-2 were found to be normally distributed. t-α-syn, Aβ42, tTau and pTau were not normally distributed and therefore were log-transformed for group comparisons. Differences in CSF contactin-1 and -2 levels between several diagnostic groups (or two diagnostic groups) were tested by the general linear model (GLM) adjusted for age and sex with the Bonferroni correction for multiple comparisons. Correlation analyses were done using the Spearman correlation test. Receiver-operating characteristic (ROC) analyses were performed to evaluate the diagnostic performance of contactins for discrimination of PD from controls and DLB. The statistical tests were two-tailed and values with *p* < 0.05 were considered significant. Statistical analyses were performed using SPSS version 22 (IBM SPSS Statistics for Windows, Version 21.0. Armonk, NY: IBM Corp). Graphs were plotted using GraphPad Prism version 6.07.

## 3. Results

The mean age of patients with PD was lower than the age of the DLB patients (*p =* 0.02). There were more males than females in every diagnostic group (See Table 1 for demographic and clinical details). Contactin-1 levels were higher in males than females in the control group (*p =* 0.01) and DLB group (*p <* 0.001), whereas contactin-2 levels were similar in males and females across all diagnostic groups.

**Figure 1:**
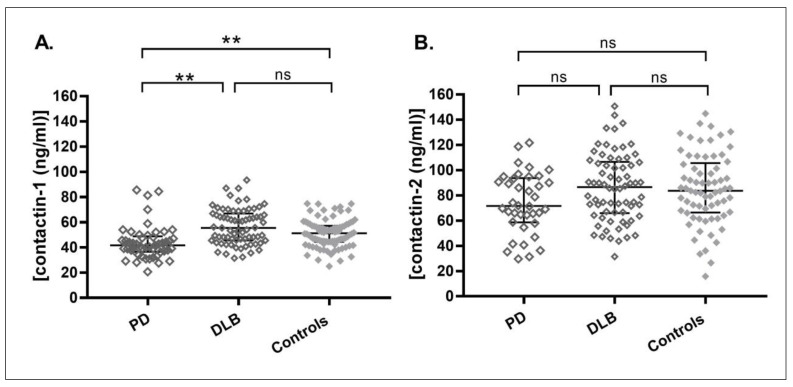
Levels of cerebrospinal fluid (CSF) contactin-1 (**A**) and contactin-2 (**B**) in PD, DLB and controls. The long horizontal line represents the median and the short horizontal lines represent the inter-quartile range (IQR), respectively. ** *p <* 0.01. The *p*-values displayed are corrected for multiple comparisons (Bonferroni correction), sex and age.

Median values and group differences of the CSF biomarkers- contactin-1, contactin-2, tTau, pTau and t-α-syn are shown in Table 1. Contactin-1 levels were lower in PD patients compared to controls (19% lower, *p =* 0.003) and DLB patients (7% lower, *p =* 0.001) (Figure 1A). Contactin-1 levels in different diagnostic groups, with the control group stratified into SCD and healthy controls, are shown in Figure S8. Next, we evaluated the performance of contactin-1 in discriminating PD from controls and DLB. Contactin-1 could not discriminate PD from controls alone (area under the curve (AUC) (confidence interval (CI)) = 0.65 (0.54–0.0.76), *p =* 0.009). Although it slightly improved the model (comprising t-α-syn, tTau and pTau) (combined AUC (CI) = 0.73 (0.62–0.82), *p =* 0.0002) (Figure S1). The combination of contactin-1 with t-α-syn, tTau, pTau and Aβ42 yielded AUC (CI) = 0.88 (0.80–0.94, *p <* 0.0001) to discriminate PD from DLB (Figure S1).

Contactin-2 levels were not significantly different after correction for multiple comparisons, sex and age (Figure 1B). In line with the weak reduction of contactin-2 in PD, AUCs were 0.61 (0.50–0.72) for PD versus controls, and 0.62 (0.51–0.73) for PD versus DLB.

**Figure 2:**
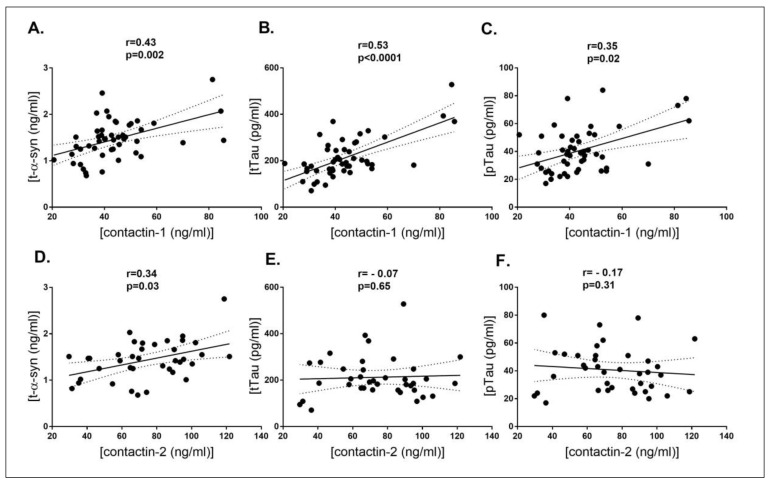
Correlation of CSF contactin-1 (**A**–**C**) and contactin-2 (**D**–**F**) with CSF t-α-syn, tTau and pTau in PD patients. Each dot in the scatter plot represents a sample. *r* = Spearman’s correlation coefficient.

Correlations of contactin-1 and -2 with commonly used CSF markers (t-α-syn, tTau and pTau) and clinical measures of disease severity such as MMSE score, disease duration, Hoehn and Yahr (H&Y) scale (only in the PD group) and Unified Parkinson’s Disease Rating Scale (UPDRS-III) (only in the PD group) were calculated within each diagnostic group. Within the PD group, contactin-1 positively correlated with t-α-syn (*r =* 0.43, *p =* 0.002), tTau (*r =* 0.53, *p <* 0.0001) and pTau (*r =* 0.35, *p =* 0.02). Contactin-2 correlated positively only with t-α-syn (*r =* 0.34, *p =* 0.03), and no correlations were found with tTau and pTau (Figure 2). Similarly to the PD group, positive correlations were seen in the control group with t-α-syn, tTau and pTau (*r =* 0.40–0.67, *p <* 0.001) and in the DLB group (*r =* 0.42–0.58, *p <* 0.001) group (Table 2). No correlations between contactin-1 and -2 levels and clinical measures of PD severity, i.e., H&Y scale (*r =* 0.002, *p =* 0.98) and UPDRS-III (*r =* 0.16, *p =* 0.39) and disease duration (*r =* −0.01, *p =* 0.93) were observed. No correlations were observed with MMSE scores (*r =* −0.009 to 0.11, *p =* 0.96 to 0.32) in any of the groups.

**Figure 3:**
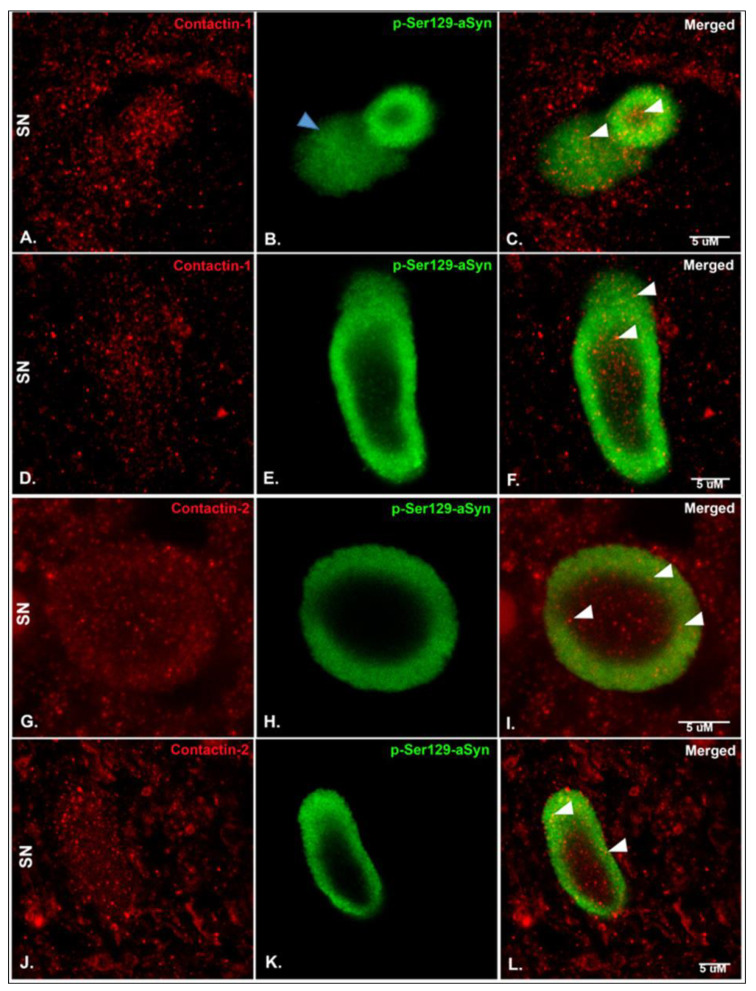
Representative photomicrographs of sections immunostained for contactin-1, contactin-2 and p-Ser129-aSyn in the substantia nigra (SN) of post-mortem human PD brain sections. (**A**–**C**) Contactin-1 shows punctate staining which co-localizes with p-Ser129-aSyn within Lewy bodies (LBs). (**D**–**F**) Contactin-1 in bulgy Lewy neurites (LNs). (**B**) A Lewy body and diffuse cytoplasmic (shown by blue arrowhead) stain of p-Ser129-aSyn is visible. (**G**–**I**) Contactin-2 similarly shows punctate staining, which co-localizes with α-syn within LBs. (**J**–**L**) Contactin-1 in bulgy LNs. White arrowheads indicate the presence of Contactin-1/-2 within LBs/LNs. Scale bar: 5 uM. Colocalization quantification is shown in Figure S6.

Considering the CSF contactin changes observed in PD patients, we next characterized contactin-1 and contactin-2 expression in post-mortem PD and control brain sections and evaluated their expression in relation to p-Ser129-aSyn, which is one of the main components of LBs and bulgy LNs [30] (Figure 3). Punctate synaptic-like staining was observed for contactin-1 and -2 in the substantia nigra (SN), hippocampus CA2 region and entorhinal cortex (Figure 3, Figures S3 and S4). Interestingly, both contactin-1 and contactin-2 were found within p-ser129-aSyn immunoreactive LBs (Figure 3A–C,G–I; shown with white arrowheads), bulgy LNs (Figure 3D–F,J–L; shown with white arrowheads), but not in thin threads (data not shown), in PD SN. The distributions of contactin-1 and contactin-2 were observed throughout LBs. However, we observed that the pattern of distribution of contactins in bulgy LNs was quite variable. Sometimes, both contactins clustered in the center of bulgy LNs and sometimes the distribution was more dispersed (Figures S3 and S4). No apparent differences in staining patterns of contactin-1 and contactin-2 between PD and controls were visible. Further synaptic-like immunoreactivity of contactin-1, observed via light microscopy, in both controls and PD brain tissue samples is shown in Figure S5. We observed a similar staining pattern for contactin-2 in controls and PD in the SN, CA2 and entorhinal cortex (data not shown).

## 4. Discussion

In this cross-sectional study, analyzing CSF levels of contactin-1 and -2 in a large cohort of patients with PD and DLB, we found that the levels of synaptic protein contactin-1 in CSF were lower in PD patients compared to controls and DLB patients, but contactin-2 levels were similar across diagnostic groups. Contactin-1 and contactin-2 levels did not correlate with disease duration, MMSE, H&Y or UPDRS-III scores of PD patients. However, contactin-1 significantly correlated with CSF t-α-syn, tTau and pTau in PD patients. Interestingly, both contactin-1 and contactin-2 were present within LBs and bulgy LNs and colocalized with α-syn pathology (Figure S6).

Our result of lowered contactin-1 levels in PD compared to controls is supported by the results of a previous proteomics-based study, where contactin-1 levels tended to be reduced in CSF of PD patients versus controls [19]. To our knowledge, alterations in CSF contactin-2 levels in PD patients have not been investigated before. However, we have previously reported lower CSF contactin-2 levels in AD patients compared to SCD patients [31]. A decreased contactin-1 level could be due to lower axonal volume, due to synaptic and axonal loss, which are well-known pathological mechanisms involved in PD pathogenesis [32]. Alternatively, the reduced levels in CSF could be due to trapping of synaptic proteins within LBs [33,34] in the brain, leading to diminished release of contactins in the CSF. We did not find similar reductions in contactin-1 levels in CSF of DLB patients like those we found in PD. We have previously shown that contactins are absent or reduced in and around amyloid plaques in AD brain tissue [31]. It can be speculated that contactin sequestration in Lewy bodies may not occur due to more abundant beta amyloid co-pathology in DLB brains. Thus, future studies should directly compare contactin expression in Lewy bodies and Lewy neurites in both PD and DLB brains.

Our findings of higher levels of contactin-1 in DLB compared to PD were in contrast to the expected reductions such as those seen in PD. This increase in contactin-1 was similar to the pattern of CSF tTau levels, which was higher in DLB compared to the other diagnostic groups and correlated with contactin-1 levels (Table 2), which may indicate that contactin-1 could be a marker for ongoing neurodegeneration in DLB.

The pattern of lower contactin-1 levels in PD compared to DLB was similar to that observed for CSF t-α-syn, where lower levels were also seen in PD compared to the other diagnostic groups [12,35,36,37]. Our correlation analyses similarly showed positive correlations of contactin-1 with t-α-syn in PD patients. Therefore, from these results, it can be speculated that contactins and t-α-syn might both be markers of synaptic protein accumulation in LBs, possibly as a consequence of synaptic loss. Interestingly, contactin-1 was positively correlated with tTau and pTau within the PD group, whereas contactin-2 was not. These correlations appeared moreover to be the strongest within controls (Figure 2 and Table 2). These results indicate that contactins may be physiologically associated with these proteins.

Next, we studied the localization of contactin-1 and contactin-2 in SN, hippocampus and entorhinal cortex of PD patients and controls, to understand if there could be a pathobiological correlate for the CSF findings. To our knowledge, this is the first study showing contactin-1 and -2 expression patterns in the PD brain. We observed a punctate synaptic-like staining in these brain areas in PD patients, as well as in non-demented controls, similar to what was previously observed in the hippocampus and temporal cortex of AD patients and non-demented controls [31]. Interestingly, we additionally found contactin expression within LBs and bulgy LNs. This finding is in accordance with recent studies that observed localization of another synaptic protein, synapsin, within LBs and LNs [33,34], indicating that these structures contain various types of vesicular entities comprising synaptic proteins, which we now extend to contactin-1 and contactin-2 proteins. Moreover, another study has similarly shown co-localization of contactin-1 and synapsin in hippocampal cell cultures, confirming the comparable localization pattern of contactins to that of synapsin [38].

Both CSF t-α-syn and contactin-1 were significantly decreased in PD patients compared to controls in our cohort, indicating that both these proteins could be sequestered together in Lewy bodies. It is known that a Lewy bodies may also show tau immunoreactivity, especially in neuronal populations vulnerable to both neurofibrillary tangles and Lewy Bodies [39,40]. In future, tau immunoreactivity should be tested along with contactin immunoreactivity within Lewy bodies to better understand the pathological relationship of contactin and tTau and pTau in PD.

The major strength of our study was that analytically validated, commercially available ELISAs were used for measuring contactin levels in CSF, leading to easier replication in larger cohorts compared to previous proteomics-based contactin studies [19]. Moreover, we used a larger sample size compared to previous studies. A limitation of our study was that even though we included 220 subjects that were well-characterized in terms of clinical data, we did not yet validate the findings in another independent cohort. Another limitation was that PD and healthy controls were obtained from one cohort, whereas DLB and SCD were obtained from another cohort. However, all these samples were collected at the same center, following exactly same protocols, excluding pre-analytical variation as a possible confounder. There was a lack of longitudinal data, which did not allow us to examine the value of contactins as predictive biomarkers. It remains to be investigated in future studies how these biomarkers differentiate PD from clinically relevant diagnostic groups such as multiple system atrophy (MSA), essential tremor, etc.

## 5. Conclusions

Our results indicate that synaptic degeneration might be reflected by CSF contactin-1 in PD patients, but its performance in clinical practice will be limited. Our results suggest the possible use of contactin-1 to complement panels for monitoring synaptic dysfunction or protection in therapeutic paradigms, rather than as a single diagnostic biomarker. Our findings further support the suggestion that synaptic proteins are affected and may start accumulating within neurons in PD. Considering that contactin levels in CSF did not vary in a similar fashion in PD and DLB, studies unraveling a mechanistic relation between contactins, α-syn, tau and Aβ are required to understand the cell signaling pathway underlying PD and DLB pathogenesis.

## Figures and Tables

**Table 1:** Demographic details of subjects.

	PD	DLB	Controls
**n**	58	72	90
**Sex (female %)**	38	10	41
**Age (years)** (mean ± SD)	63 ± 10 ^e^	68 ± 6 ^c^	64 ± 7
**MMSE** (mean ± SD)	29 ± 2	23 ± 4 ^a,d^	29 ± 1
**Disease duration** (median(IQR))	4 (2–10)	2.5 (2.0–4.0)	–
**H&Y** (mean ± SD)	2 ± 0.5	–	–
**UPDRS-III** (mean ± SD)	23 ± 9	–	–
**tTau (pg/mL)** (median(IQR))	190 (157–274)	306 (224–369) ^a,d^	229 (174–272)
**pTau (pg/mL)** (median(IQR))	40 (28–51)	47 (35–61)	44 (34–50)
**Aβ42 (pg/mL)** (median(IQR))	967 (794–1076)	710 (560–937) ^a,d^	1009 (848–1139)
**Contactin-1(ng/mL)** (median(IQR))	42 (36–49) ^b^	56 (46–67) ^e^	52 (45–58)
**Contactin-2 (ng/mL)** (median(IQR))	72 (59–94)	87 (66–106)	84 (66–106)
**t-α-syn (pg/mL)** (median(IQR))	1.47 (1.25–1.77) ^c^	1.40 (1.10–1.70) ^b^	1.71 (1.40–1.93)

MMSE, Mini Mental State Examination; H&Y, Hoehn and Yahr; UPDRS-III, Unified Parkinson’s Disease Rating Scale. ^a^ *p <* 0.001 dementia with Lewy bodies (DLB)/Parkinson’s disease (PD) versus controls, ^b^ *p <* 0.01 DLB/PD versus controls, ^c^ *p <* 0.05 DLB/PD versus controls; ^d^ *p <* 0.001: PD versus DLB, ^e^ *p <* 0.01: PD versus DLB.

**Table 2:** Correlations between contactins and other CSF biomarkers in DLB and control groups.

	DLB		Controls	
	Contactin-1	Contactin-2	Contactin-1	Contactin-2
**t-α-syn**	*r =* 0.55 *p* = 0.001	*r =* 0.26 *p* = 0.13	*r =* 0.66 *p* < 0.001	*r =* 0.41 *p* = 0.02
**tTau**	*r =* 0.55 *p* < 0.001	*r =* 0.46 *p* < 0.001	*r =* 0.62 *p* < 0.001	*r =* 0.44 *p* < 0.001
**pTau**	*r =* 0.58 *p* < 0.001	*r =* 0.43 *p* < 0.001	*r =* 0.67 *p* < 0.001	*r =* 0.50 *p* < 0.001

*r =* Spearman’s correlation coefficient.

## Supplementary Materials

The following are available online at https://www.mdpi.com/2218-273X/10/8/1177/s1, Figure S1: Area under receiver-operating characteristic (ROC) curves for contactin-1 for discriminating PD from controls (A) and PD from DLB (B). Figure S2: Representative photomicrographs of negative controls (substantia nigra (SN)). (A,D) were immunolabelled with only donkey anti-rabbit alexa-594 secondary antibody (contactin primary antibodies were omitted). (B,E) were immunolabelled for p-Ser129-aSyn with subsequent addition of the corresponding secondary antibody; (C,F) shows the merged images of red and green channels. Contactin-1- and contactin-2-specific signals cannot be seen (A,D) in the absence of contactin primary antibodies, indicating the absence of non-specific signals from the secondary antibody only. Scale bar: 5 uM. Figure S3: Representative photomicrographs of sections immunostained for contactin-1 and p-Ser129-aSyn in the substantia nigra (SN) (A–C), hippocampus CA2 region (D–F) and entorhinal cortex (ENT) (G–I) of post-mortem human PD brain sections. The distribution of contactin-1 was seen throughout the bulgy Lewy neurites, whereas in others the distribution pattern was more clustered. Figure S4: Representative photomicrographs of sections immunostained for contactin-2 and p-Ser129-aSyn in the substantia nigra (SN) (A–C), hippocampus CA2 region (D–F) and entorhinal cortex (ENT) (G–I) of post-mortem human PD brain sections. The pattern of contactin-2 expression is different in different types of Lewy neurites. Figure S5: Representative photomicrographs of sections immunostained for contactin-1 in the substantia nigra (SN) (A,B), hippocampus CA2 region (C,D) and entorhinal cortex (ENT) (E,F) of post-mortem human control (left panel) and PD (right panel) brain sections. Synaptic-like punctate contactin-1 expression can be seen in the extracellular matrix, cell body, nucleus and axonal processes (shown with black arrowheads). Possible neuromelanin-positive cells in the SN are shown with blue arrowheads. Figure S6: (A) Colocalization quantification using ‘colocalization threshold’ plug-in in ImageJ-Fiji. M1, M2 are Mander’s coefficients and tM1, tM2 are thresholded Mander’s coefficients. The bar plots represent mean±SD values of Mander’s coefficients for all images quantified (n = 6 images quantified per group). The overall Pearson’s correlation coefficient ranged between 0.1 and 0.4. (B) Representative photomicrographs of sections immunostained for contactin-1 and contactin-2. The rightmost panels show the colocalized signal above the threshold (in white). Figure S7: Levels of CSF contactin-1 (A), contactin-2 (B), normalized Aβ42 (C), normalized tTau (D), normalized pTau (E) and MMSE scores (F) in controls and SCD. The long horizontal line represents the median and the short horizontal lines represent the inter-quartile range (IQR), respectively. The *p*-values displayed are corrected for sex and age. Figure S8: Levels of CSF contactin-1 in PD, DLB, SCD and healthy controls. The long horizontal line represents the median and the short horizontal lines represent the inter-quartile range (IQR), respectively. * *p* < 0.05, ** *p* < 0.01. The *p*-values displayed are corrected for multiple comparisons (Bonferroni correction), sex and age.

## Appendix A

### Appendix A.1. Clinical Diagnosis

PD patients were diagnosed according to the United Kingdom Parkinson’s Disease Society Brain Bank (UK-PDSBB) clinical diagnostic criteria; data were collected in 2010 by movement disorders specialists [41]. The high Mini Mental State Examination (MMSE) (>28) scores of PD patients did not indicate dementia. Disease duration was defined as the time-period between the first subjective complaints of motor symptoms and the time of lumbar puncture. UPDRS-III [42] and the modified H&Y [43] classification were used for rating the severity of Parkinsonism and disease stage in the ‘on’ state. Patients with DLB were diagnosed based on the 2005 consensus criteria and additionally fulfilled new consensus criteria [44,45]. Subjects were labeled as SCD when cognitive complaints could not be confirmed by cognitive testing and criteria for mild cognitive impairment, dementia or any other neurological or psychiatric disorder known to cause cognitive complaints were not fulfilled. SCD patients were included in this study when they remained cognitively stable for at least two years and dementia could be excluded using extensive neuropsychological testing. Healthy volunteers underwent a standardized clinical assessment that included medical history and neurological examination. Dementia was excluded in healthy controls using the Cambridge Cognitive Examination (CAMCOG) scale [46].

### Appendix A.2. Post-Mortem Brain Tissue

Formalin-fixed paraffin-embedded post-mortem substantia nigra (SN) and hippocampal tissue of clinically diagnosed and pathologically confirmed PD patients (*n* = 4) and age-matched non-demented controls (*n* = 4) were obtained from the Netherlands Brain Bank (NBB; www.brainbank.nl). Written informed consent for brain autopsy and use of medical records for research purposes were obtained from all donors or their next of kin. For pathological assessment, staining of selected regions was performed using hematoxylin and eosin, Gallyas silver stain and immunohistochemistry against α-synuclein (clone KM51, 1:500, Monosan, Uden, The Netherlands), amyloid-β (clone 6f/3d, 1:100, DAKO, Amstelveen, The Netherlands) and hyperphosphorylated tau (clone AT8, 1:100, Innogenetics, Ghent, Belgium). For pathological staging of α-synuclein, amyloid-β, neurofibrillary and neuritic plaque pathology, diagnostic criteria were used according to the BrainNet Europe and the National Institute on Aging-Alzheimer’s Association guidelines [47,48,49]. Patient details, such as clinical and pathological diagnosis, age, sex and post-mortem delay, are outlined in Table A1.

**Table A1:** Clinical details of patients included in the post-mortem study.

Patient Number	Clinical Diagnosis	Pathological Diagnosis	Sex	Age	Braak NFT Stage	CERAD Amyloid	Braak Alpha-Synuclein Stage	Post Mortem Delay (Hours:Minutes)	pH of CSF	Brain weight (g)	Disease Duration (years)	Cause of Death
1	PD	PD	F	76	1	O	6	5:30	6.16	1248	15	Aspiration pneumonia, cardiac arrest
2	PDD	PDD	M	80	1	O	6	5:25	6.43	1180	13	Aspiration pneumonia
3	PDD	PDD	M	80	2	B	6	5:30	6.29	1279	13	
4	PDD	PDD	M	72	1	A	6	4:00	6.22	1210	8	
5	Control	Control	F	85	3	A	0	6:25	6.60	1080	NA	Euthanasia, ischemic changes in F2
6	Control	Control	M	89	2	O	0	6:50	6.23	1185	NA	Urosepsis
7	Control	Control	M	80	1	B	0	7:00	6.22	1354	NA	Dehydration by epilepsy by brain tumor
8	Control	Control	F	78	1	A	0	7:10	6.32	1120	NA	

## Appendix B

### Appendix B.1. Immunohistochemistry (IHC), Immunofluorescence (IF) and Microscopy

Formalin-fixed and paraffin-embedded brain tissue was cut into 10-μm-thick sections and processed for immunohistochemistry. All sections were baked at 56 °C on a heated plate for 60 min. Deparaffinization was carried out in 100% xylene substitute and decreasing concentrations of ethanol. Antigen retrieval was performed by steaming (98 °C) for 30 min in 10 mM citrate buffer at pH 6.0. Blocking was done using 2% normal donkey serum in tris buffer saline before adding the primary antibodies for overnight incubation at 4 °C. The sections were washed with TBS between incubation steps. Anti-contactin-1 (1:100, cat no. ab66265, Abcam, Cambridge, UK) and anti-Contactin-2 (1:100, HPA001397, Atlas Antibodies, Stockholm, Sweden) were used as primary antibodies for IHC. Labelling of primary antibody was detected using DAKO anti-rabbit/mouse EnVision+ System-HRP (DAKO, 45007, Glostrup, Denmark). Nuclei were visualized by Mayer’s hematoxylin counterstain (Merck, MHS1, Zwijndrecht, The Netherlands). Finally, the slides were dehydrated with increasing series of ethanol solution and mounted with coverslips (Thermo Fisher Scientific Gerhard Menzel B.V. & Co. KG, Braunschweig, Germany) using Entellan mounting medium (cat no. 107960, EMD Millipore, Massachusetts, United States). Transmitted light microscopy images were taken using a Leica DM500 microscope with a 20× and 40× oil lens (HC PL-APO, numerical aperture 1.40) and Leica Application Suite version 44 software.

The following primary antibodies were used for evaluation of colocalization using IF between contactin-1/2 and pathological α-synuclein: rabbit polyclonal anti-contactin-1 (1:40; cat no. ab66265, Abcam, Cambridge, UK), rabbit polyclonal anti-contactin-2 (1:40; HPA001397, Atlas Antibodies, Stockholm, Sweden) in combination with mouse monoclonal anti-p-Ser129-aSyn (11A5 1:30,000, courtesy of Prothena Biosciences [29]. Donkey anti-rabbit IgG Alexa-594 (1:200, cat no. A-21207, Thermo Fisher Scientific, Landsmeer, Netherlands) and donkey anti-mouse IgG Alexa-488 (1:200; A21202, Mol. Probes, Thermo Fisher Scientific) were used as the secondary antibody. Slides were mounted with coverslips using Mowiol as a mounting medium. LBs and LNs were first identified in neurons in the SN, CA2 and entorhinal cortex of PD cases using p-α-syn signal in the immunostained sections. Next, the contactin-1 or -2 signal was evaluated. Negative controls lacking primary antibodies were performed to control for background/autofluorescence levels and aspecific staining.

Immunofluorescent images were acquired using a Leica TCS SP8 STED 3X microscope (Leica Microsystems) with HC PL-APO CS2 100× 1.4 NA oil objective lens, with a 1024 × 1024 pixel format for the images. All signals were detected using gated hybrid detectors in counting mode. Sections were sequentially scanned for each fluorophore, by irradiation with a pulsed white light laser at different wavelengths (488 or 594). Stacks in the Z-direction were made for each image. To obtain CSLM images of the DAPI signal, sections were irradiated with a solid-state laser at a wavelength of 405 nm. Final figures were composed using Adobe Photoshop (CS6, Adobe Systems Incorporated). Negative controls (without contactin-1 or contactin-2 primary antibody) are shown in Figure S2.
