# Supplementary material for: Contactin-1 Is Reduced in Cerebrospinal Fluid of Parkinson’s Disease Patients and Is Present within Lewy Bodies
Source: Biomolecules. 2020 Aug 12;10(8):1177. doi: 10.3390/biom10081177 (PMC7463939; doi:10.3390/biom10081177)
Supplement: Supplementary file 1 [file biomolecules-10-01177-s001.pdf]

Supplementary Figures

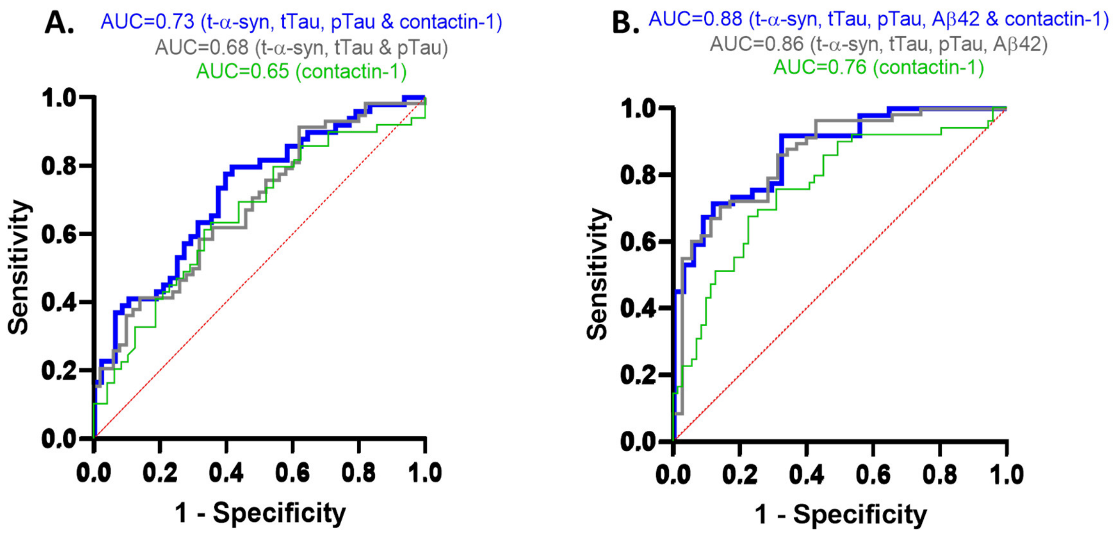

Figure S1. Area under receiver-operating characteristic (ROC) curves for discriminating PD from controls (**A**) and PD from DLB (**B**).

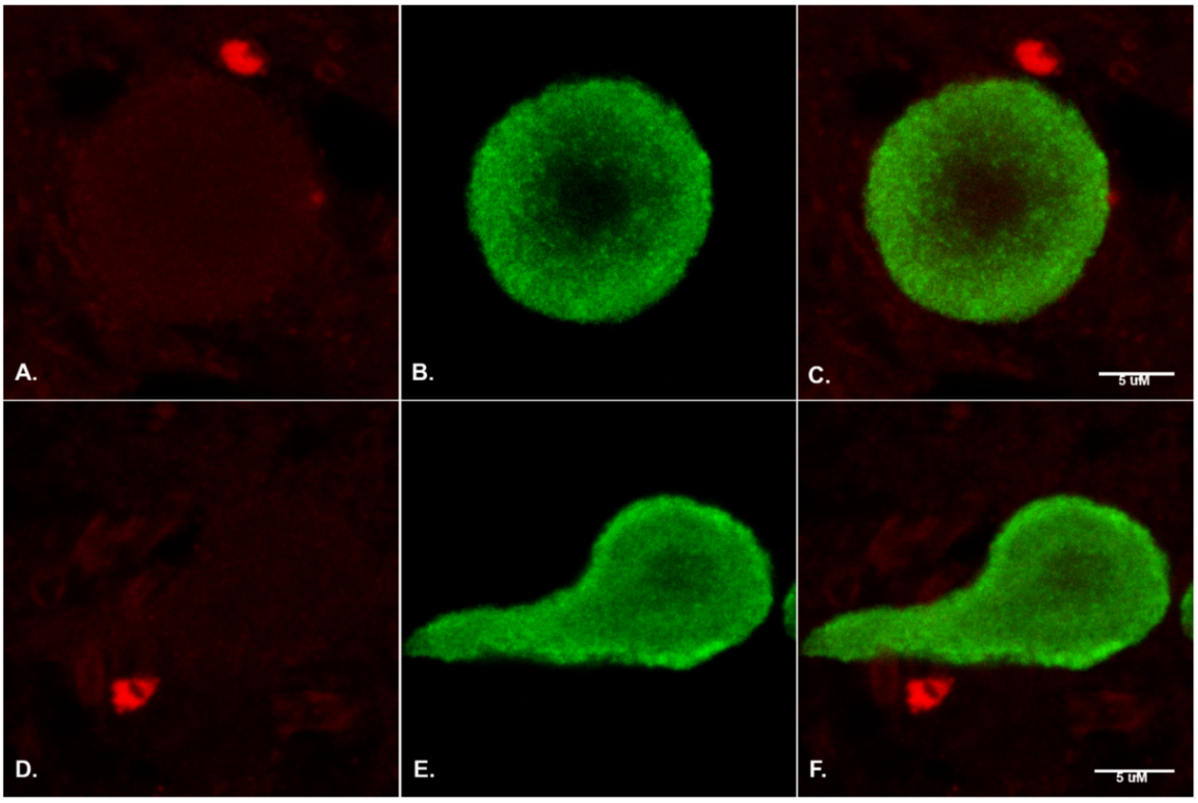

Figure S2. Representative photomicrographs of negative controls (substantia nigra (SN)). (**A**,**D**) were immunolabelled with only donkey anti-rabbit alexa-594 secondary antibody (contactin primary antibodies were omitted). (**B**,**E**) were immunolabelled for p-Ser129-aSyn with subsequent addition of the corresponding secondary antibody; (**C**,**F**) shows the merged images of red and green channels. Contactin-1 and contactin-2 specific signals cannot be seen (**A**,**D**) in the absence of

contactin primary antibodies, indicating the absence of non-specific signals from the secondary antibody only. Scale bar: 5 uM.

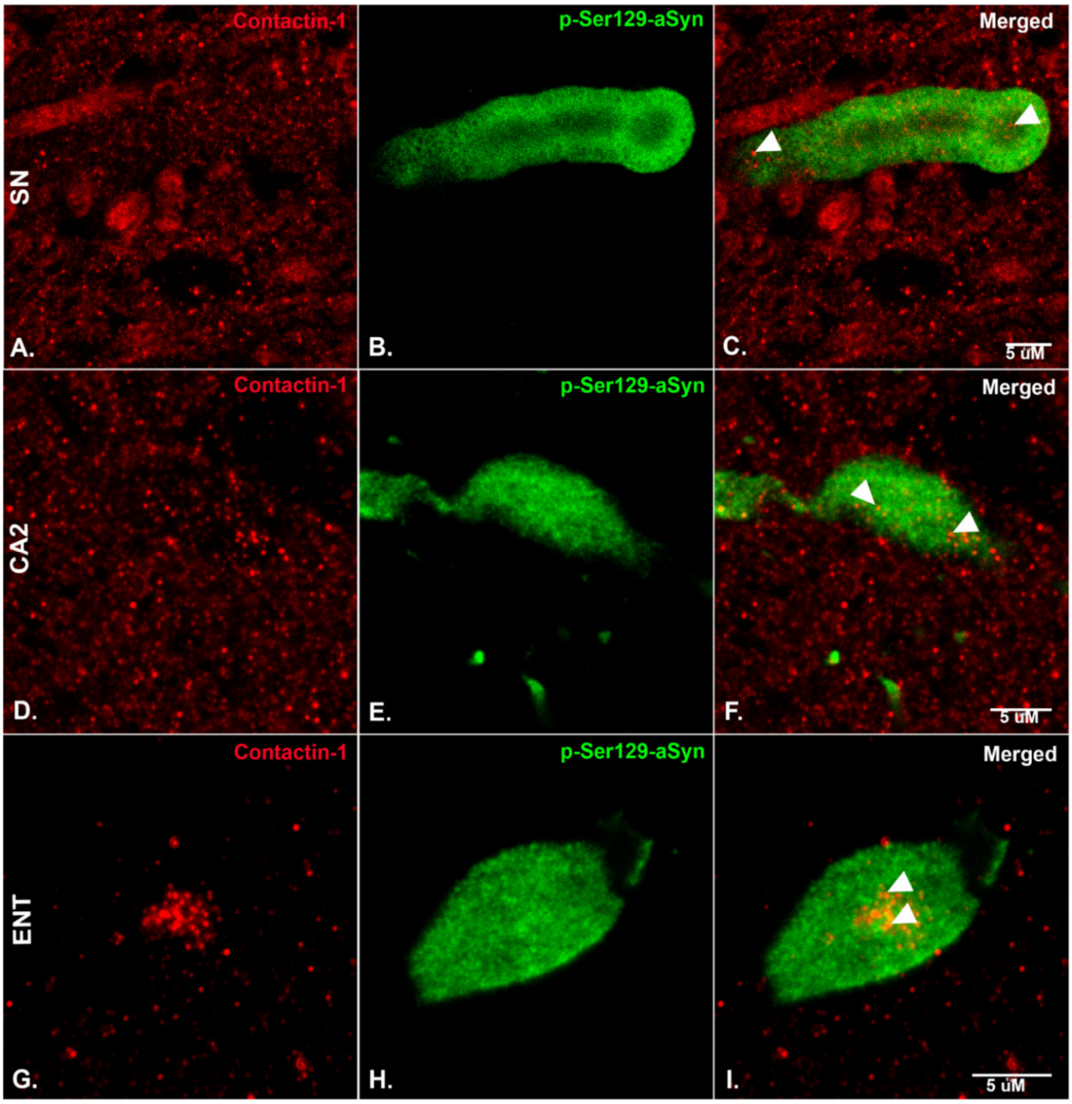

Figure S3. Representative photomicrographs of sections immunostained for contactin-1, and p-Ser129-aSyn in the substantia nigra (SN) (**A–C**)**,** hippocampus CA2 region (**D–F**) and entorhinal cortex (ENT) **(G–I)** of post-mortem human PD brain sections. The distribution of contactin-1 was seen throughout the bulgy Lewy neurites, whereas in others the distribution pattern was more clustered.

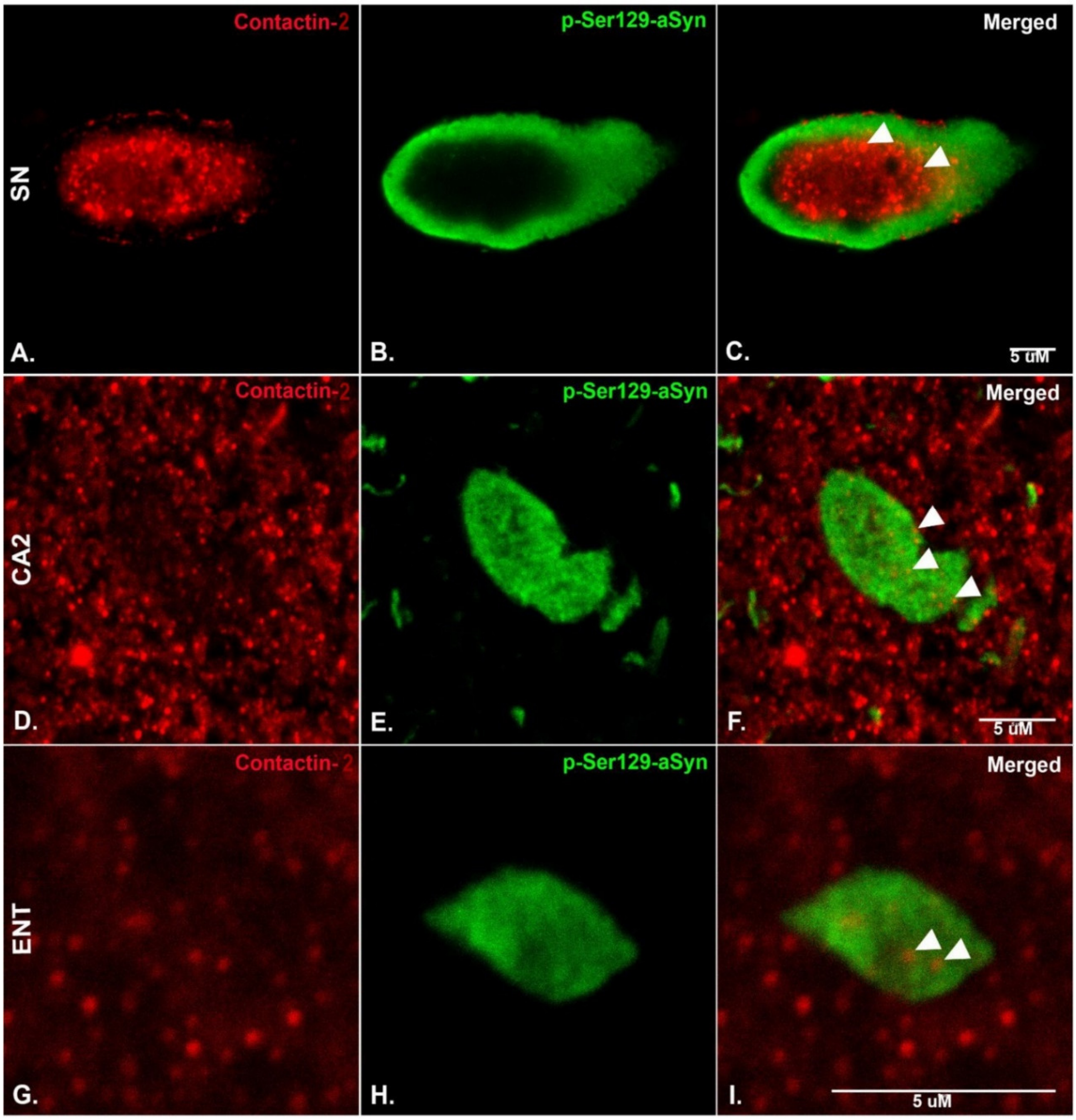

Figure S4. Representative photomicrographs of sections immunostained for contactin-2 and p-Ser129-aSyn in the substantia nigra (SN) (**A**–**C**), hippocampus CA2 region (**D**–**F**) and entorhinal cortex (ENT) (**G**–**I**) of post-mortem human PD brain sections. The pattern of contactin-2 expression is different in different types of Lewy neurites.

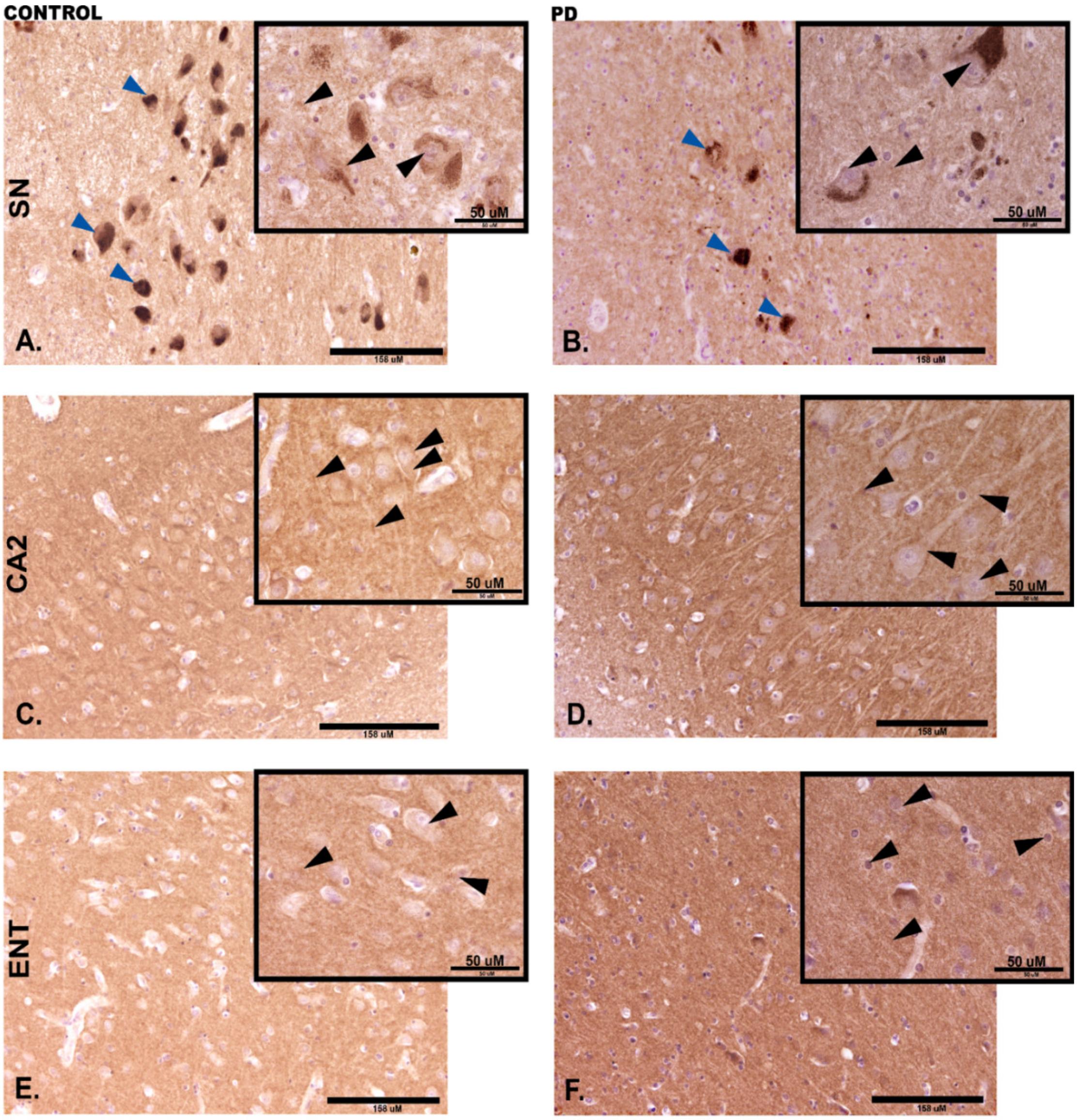

Figure S5. Representative photomicrographs of sections immunostained for contactin-1 in the substantia nigra (SN) (**A**,**B**), hippocampus CA2 region (**C**,**D**) and entorhinal cortex (ENT) (**E**,**F**) of post-mortem human control (left panel) and PD (right panel) brain sections. Synaptic-like punctate contactin-1 expression can be seen in the extracellular matrix, cell body, nucleus and axonal processes (shown with black arrowheads). Possible neuromelanin-positive cells in the SN are shown with blue arrowheads.

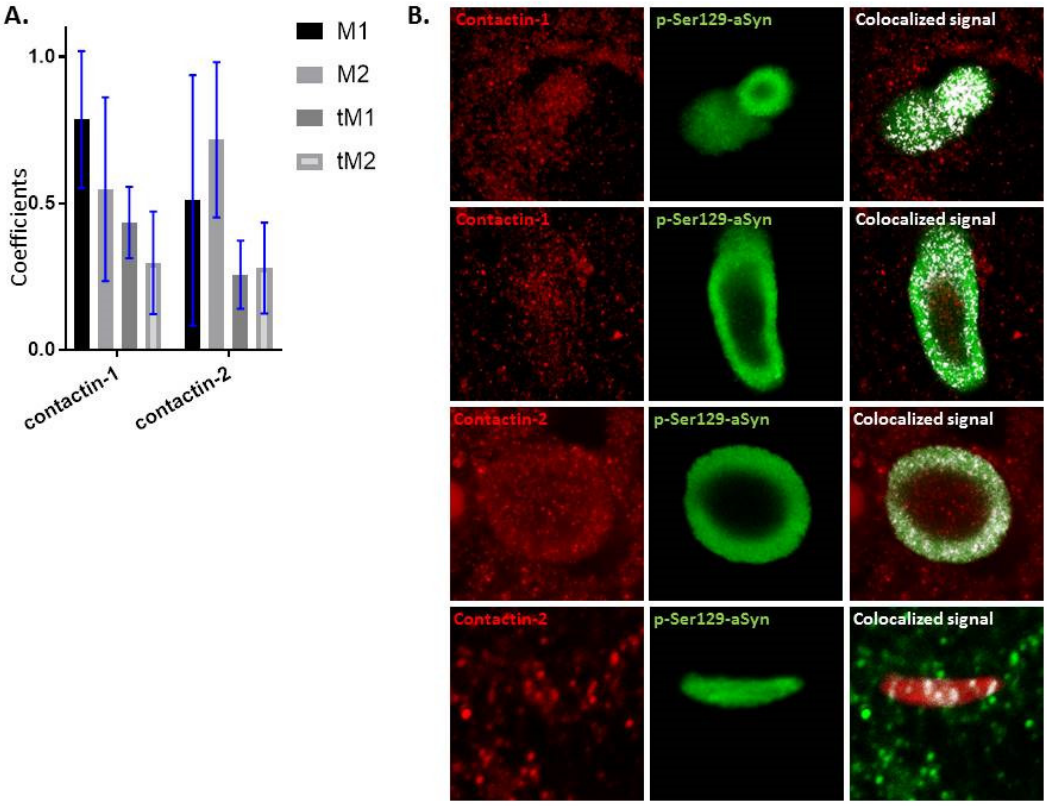

Figure S6. (**A**) Colocalization quantification using 'colocalization threshold' plug-in in ImageJ-Fiji. M1, M2 are Mander's coefficients and tM1, tM2 are thresholded Mander's coefficients. The bar plots represent mean±SD values of Mander's coefficients for all images quantified (*n* = 6 images quantified per group). The overall Pearson's correlation coefficient ranged between 0.1 and 0.4. (**B**) Representative photomicrographs of sections immunostained for contactin-1 and contactin-2. The rightmost panels show the colocalized signal above the threshold (in white).

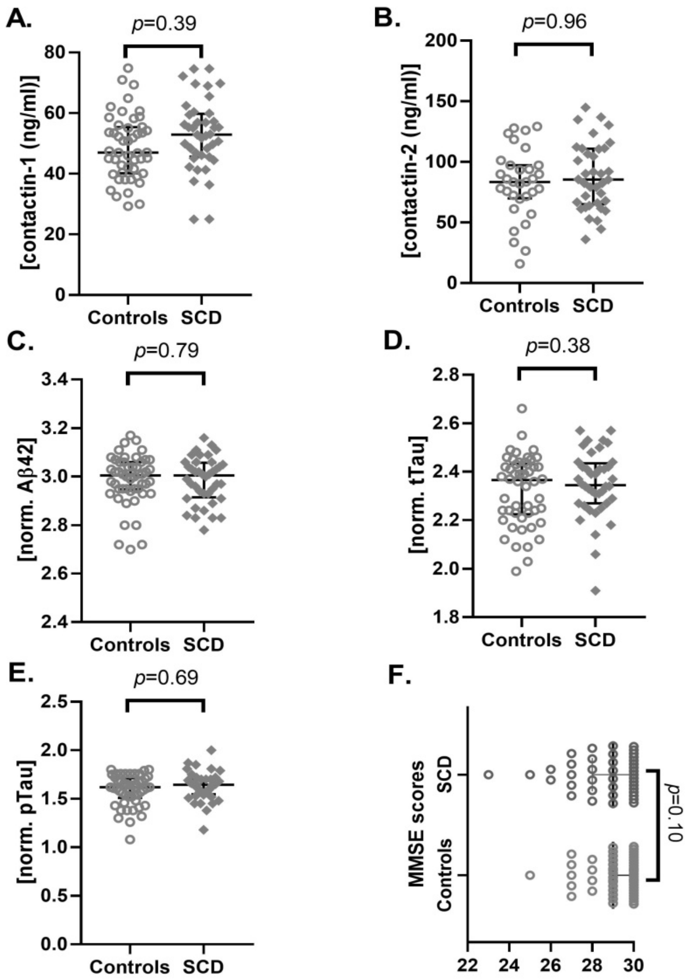

Figure S7. Levels of CSF contactin-1 (**A**), contactin-2 (**B**), normalized Aβ42 (**C**), normalized tTau (**D**), normalized pTau (**E**) and MMSE scores (**F**) in controls and SCD. The long horizontal line represents median and the short horizontal lines represent inter-quartile range (IQR) respectively. The *p*-values displayed are corrected for sex and age. .

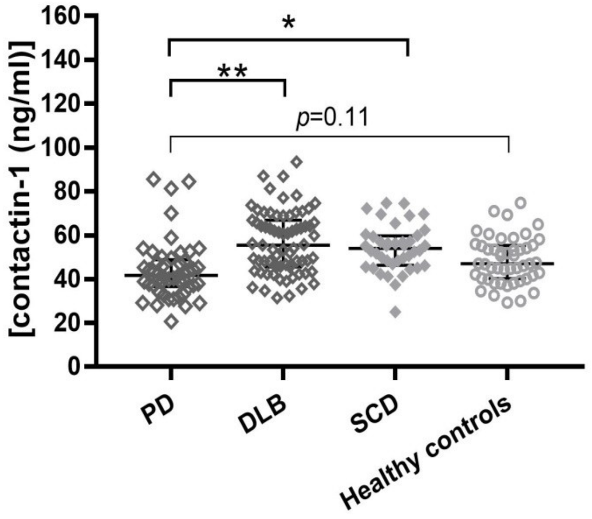

Figure S8. Levels of CSF contactin-1 in PD, DLB, SCD and healthy controls. The long horizontal line represents the median and the short horizontal lines represent the inter-quartile range (IQR),

respectively. * $p < 0.05$, ** $p < 0.01$. The p-values displayed are corrected for multiple comparisons (Bonferroni correction), sex and age.
